# Transient Lactic Acidosis and Elevation of Transaminases after the Introduction of Remdesivir in a Patient with Acute Kidney Injury

**DOI:** 10.1155/2024/6631866

**Published:** 2024-02-22

**Authors:** Elise André, Florian Lemaitre, Marie-Clémence Verdier, Vincent Haufroid, João Pinto Pereira, Philippe Hantson

**Affiliations:** ^1^Department of Intensive Care, Cliniques Saint-Luc, Université Catholique de Louvain, 1200 Brussels, Belgium; ^2^Univ Rennes, CHU Rennes, Inserm, EHESP, Irset (Institut de recherche en santé, Environnement et travail)-UMR_S 1085, Rennes, France; ^3^FHU SUPORT, Rennes F-35000, France; ^4^Clinical Chemistry Department, Cliniques universitaires Saint-Luc, 1200 Brussels, Belgium; ^5^Louvain Centre for Toxicology and Applied Pharmacology, UCLouvain, 1200 Brussels, Belgium

## Abstract

A 56-year-old woman was transferred to the intensive care unit (ICU) two days after an allogeneic stem cell transplantation (ASCT) when she presented acute respiratory distress due to the relapse of a SARS-CoV-2 infection. Following that, she received two intravenous doses of 100 mg remdesivir. Subsequently, the patient developed multiple instances of diarrhea, progressing to oliguria and acute kidney injury, necessitating continuous venovenous hemofiltration (CVVH). Despite the absence of signs of hypoxemia or cardiocirculatory failure requiring vasopressor intervention, a progressive lactic acidosis emerged. Two days after the onset of lactic acidosis, a significant rise in aminotransferases and lactate dehydrogenase occurred, in the absence of encephalopathy and coagulation disorders. Remdesivir therapy had been interrupted upon the initial signs of lactic acidosis. Despite an improvement in liver function tests and lactic acidosis, the patient's condition deteriorated, ultimately leading to her demise on day 29 due to newly arising hematological complications.

## 1. Introduction

Remdesivir is a nucleoside analogue prodrug originally developed against Ebola virus before receiving formal approval for the treatment of hospitalized patients suffering from COVID-19. While a variable proportion of COVID-19 patients, primarily those in a critical condition, may develop some form of hepatocellular or liver injury, the contribution of medications, including remdesivir, requires further exploration. In this context, we present a recent case of transient lactic acidosis and elevation of transaminase levels that occurred shortly after initiating remdesivir in a COVID-19 patient with impaired renal function. We discuss potential mechanisms and provide toxicokinetic data for remdesivir and its metabolite, GS-441524.

## 2. Case Presentation

**Figure 1:**
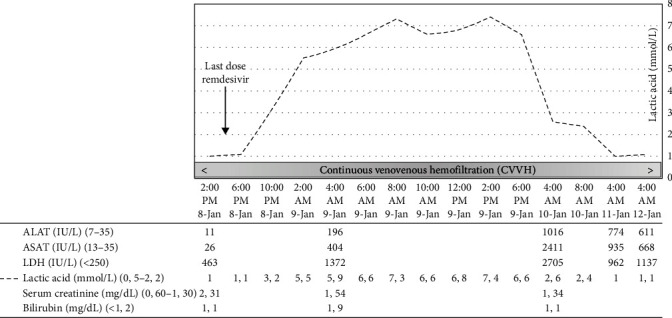
Evolution of laboratory investigations after the last administration of remdesivir.

A 56-year-old woman had a medical history of peripheral T-cell lymphoma (grade IV according to Ann Arbor classification) which was diagnosed in August 2020. She underwent an autologous stem cell transplant in January 2021. Unfortunately, a relapse of T lymphoma occurred in August 2022. For this relapse, she underwent a second cycle of chemotherapy, with an allogeneic stem cell transplantation (ASCT) planned for January 5, 2023. On December 26, 2022, the patient's nasopharyngeal swab tested positive for SARS-CoV-2 with 12.512.603,14 copies. Her respiratory symptoms were limited to a sore throat and nose congestion. Notably, she had previously declined COVID-19 vaccination. As a therapeutic measure, she received nirmatrelvir (600 mg/day) and ritonavir (200 mg/day) over a span of five days, from December 26 to 30. On December 30, prophylactic anti-infectious measures were initiated, encompassing the administration of fluconazole and acyclovir. Additionally, as a preemptive measure to address potential febrile neutropenia in anticipation of the upcoming ASCT scheduled for January 5, empiric treatment with piperacillin/tazobactam was commenced on January 2. Notably, on that day, the patient's nasopharyngeal swab showed a weakly positive result for SARS-CoV-2 with <1000 copies. Due to the limited clinical impact of the SARS-CoV-2 infection, which was also improving, as evidenced by the PCR test (02/01) and the CT scan conducted on January 5, revealing COVID sequelae with lesions resolving compared to the comparative scan from the 25th of December, there was no reason to postpone the ASCT. As planned, the patient underwent ASCT on January 5, 2023; her immunosuppressive therapy comprised tacrolimus and mycophenolic acid. On January 6, the second day post-ASCT, a deterioration in her respiratory condition led to her transfer to the intensive care unit (ICU). This coincided with an increased count of SARS-CoV-2 copies. Indeed, on that day, the patient's nasopharyngeal swab tested positive for SARS-CoV-2 with 137,415,408.73 copies. The chest X-ray realised on the 7th of January showed the appearance of extensive bilateral pulmonary opacities, predominantly in the perihilar region. Two doses of 100 mg intravenous remdesivir were administered on January 7 and 8 without an initial loading dose. After experiencing multiple episodes of diarrhea, the patient's condition progressed to oliguria and subsequently led to the development of acute kidney injury, with a calculated creatinine clearance of 1.86 mL/min. The serum tacrolimus concentration was 13.7 ng/mL (4.0-13.0). Continuous venovenous hemofiltration (CVVH) was initiated on January 7 at 4:00 pm. Due to the deterioration of respiratory failure, orotracheal intubation became necessary. Echocardiography revealed preserved left ventricular function and no decrease in central venous oxygen saturation (ScvO_2_ 62.9%). The patient developed increasing lactic acidosis even in the absence of cardiocirculatory failure or the requirement for vasopressors. Additionally, hypoglycemia was observed together with ketonuria. Two days after the onset of lactic acidosis, a peak in alanine transferase (ALT), aspartate aminotransferase (AST), and lactic dehydrogenase (LDH) levels occurred. Importantly, coagulation factors remained intact, and no signs of encephalopathy were observed (Figure 1). Liver ultrasound findings did not reveal any evidence of liver disease. Suspecting drug toxicity, the administration of remdesivir was definitely discontinued on January 8 at 5:00 pm, just after the second dose. As a result, the lactic acidosis showed gradual improvement.

The medications administered before the onset of lactic acidosis and abnormalities in liver test results are listed in Table 1.

The subsequent clinical course was marked by a deterioration of her respiratory condition and the emergence of new hematological complications, including thrombotic microangiopathy. The patient passed away on ICU day 29.

A first blood sample was collected 17 h after the final remdesivir administration. This process was repeated twice, with a 12-hour interval between collections. Remdesivir plasma concentrations were found to be below the limit of quantification of the employed method. In contrast, GS-441524 plasma concentrations were measured at 207, 159, and 102 ng/mL for the respective samples. The elimination half-life of plasma GS-441524 was calculated to be 31.5 h under CVVH therapy.

Furthermore, a genotyping analysis revealed that the patient's genetic profile included the alleles CYP2C19∗1/∗17 and SLCO1B1∗37/∗37.

## 3. Discussion

Remdesivir is a diastereomer monophosphophoramidate of the adenine nucleoside analogue GS-441524. It undergoes metabolism through carboxylesterase 1 (CES1), cathepsin A, and cytochrome P450 enzymes (CYP2C8, CYP2D6, and CYP3A4). *In vitro*, remdesivir acts as a substrate for organic anion transporting polypeptide 1B1 (OATP1B1) and P-glycoprotein (P-gp) transporters [1]. The specific metabolism of GS-441524 leading to the formation of nucleoside triphosphate (GS-443902), the active metabolite, is based on a series of intracellular phosphorylation steps. In healthy volunteers, renal excretion of a remdesivir dose constitutes approximately 10% as an unchanged drug and around 50% as GS-441524 [2]. The half-life is about 0.89 hours for remdesivir and 25 hours for GS-441524 [3].

Limited data exist regarding the pharmacokinetics of remdesivir and GS-441524 in cases of acute kidney injury. Earlier reports have indicated that GS-441524 might not be efficiently eliminated when the estimated glomerular filtration rate (eGFR) is low [2–6]. Remdesivir is not recommended for use in adults with an estimated glomerular filtration rate below 30 mL/min. One of the reasons for this recommendation is that the solubilizing excipient, sulfobutylether-beta-cyclodextrin (SBECD), is eliminated through the renal route and could potentially accumulate in patients with impaired renal function. Notably, patients with impaired renal function exhibited higher GS-441524 plasma concentrations, underscoring the significance of renal excretion as a major elimination pathway [3]. In a study involving 37 Japanese patients with renal dysfunction, it was observed that the GS-441524 trough concentrations were significantly higher in the group with an eGFR less than 60 mL/min compared to the group with an eGFR greater than or equal to 60 mL/min. However, no significant correlation was identified between serum GS-441524 concentration and the occurrence of adverse effects [4]. Similarly, a retrospective analysis of COVID-19 patients also indicated a relatively safe profile of remdesivir in patients with an eGFR below 30 mL/min [7].

In this observation, the first plasma determination of remdesivir and its metabolite GS-441524 was performed 17 h after the last administration. As expected, remdesivir was no longer detectable based on its short half-life. At the same time, GS-441524 concentrations were high even without any loading dose and exceeded the trough concentration expected in healthy subjects. Additionally, the terminal plasma elimination half-life was marginally increased (31.5 h vs. 25 h), further confirming the heightened exposure to GS-441524 in patients with renal impairment [5, 8].

The impact of renal replacement therapy on the pharmacokinetics of remdesivir and GS-441524 has been studied in a small number of patients [2, 5, 6]. The influence of interventions such as intermittent hemodialysis (IHD) or continuous renal replacement therapy is modest.

Transient and mild elevations of ALT and AST were observed during multiple-dose phase 1 studies in healthy volunteers and also in treated COVID-19 patients [8, 9]. Remdesivir was also identified as a potential contributing factor to hepatocellular injury in a few cases of COVID-19 patients [10]. In two cases of suspected remdesivir-associated acute liver failure (ALF), the administration of intravenous N-acetylcysteine (NAC) using the same protocol for acetaminophen-induced hepatotoxicity resulted in clinical and biological improvements. However, this improvement was also associated with remdesivir discontinuation, and insufficient strong evidence supports using NAC in this context [11]. Moreover, in a double-blinded, placebo-controlled, randomized clinical trial involving 83 patients, prophylactic use of NAC prevented liver transaminase elevation and liver injury in critically ill COVID-19 patients [12]. In our observation, a close temporal relationship was noted between the elevation of serum lactate and ALT levels, and the direct involvement of SARS-CoV-2 infection appeared unlikely.

Regarding drug-drug interactions, no interactions were identified between remdesivir and specific medications, such as fluconazole or tacrolimus. However, interactions with P-gp inhibitors could increase the risk of hepatotoxicity [13]. Among the medicines coadministered to the patient, tacrolimus, fluconazole, and pantoprazole are potential P-gp inhibitors, with tacrolimus and pantoprazole also exhibiting inhibition of OATP1B1. Additionally, *in vitro* studies have demonstrated a mild inhibitory effect of tacrolimus on CES1 [14].

There are obviously numerous causes of lactic acidosis in critically ill patients, and type A lactic acidosis is the far most common form, often related to impaired general or local tissue perfusion or poor oxygen delivery. In the present observation, we were able to exclude any patent cause of shock, and the patient did not experience major hypoxic episodes during mechanical ventilation. The changes in liver enzymes also followed the elevation of serum lactate concentration, excluding the role of reduced lactate hepatic clearance. Therefore, type B lactic acidosis due to mitochondrial toxicity was suspected and further supported by the development of hypoglycemia and ketonuria. Remdesivir and its metabolites have demonstrated weak inhibitory effects on human mitochondrial RNA polymerase, suggesting a potential for mitochondrial toxicity. However, experimental studies have yielded conflicting findings. In a study involving human-derived HepG2 liver cells exposed to increasing remdesivir concentrations, Bjork and Wallace found no discernible impact on mitochondrial respiratory function [15]. Minimal *in vitro* effects of remdesivir on mitochondrial respiration were observed in isolated mitochondria [16]. Conversely, in a model using human-induced pluripotent stem cell-derived cardiomyocytes, remdesivir inhibited mitochondrial respiration at levels below the estimated plasma concentrations under both normoxic and hypoxic conditions [17]. Moreover, *in vitro* mitochondrial toxicity of remdesivir in cardiac cells was also evident in a significant decrease in oxygen consumption [18].

Finally, the potential influence of genetic variants on remdesivir-associated liver enzyme elevation has also been investigated. Individuals with the CYP2C19 intermediate or poor metabolizer phenotype exhibited higher peak ALT levels than those with normal, rapid (as in our patient), or ultrarapid metabolizer phenotypes [19].

## 4. Conclusion

We have presented a case involving a patient who experienced transient lactic acidosis and elevated liver enzyme levels after the initiation of remdesivir for the treatment of SARS-CoV-2 in the context of acute kidney injury. Literature findings have demonstrated the possibility of GS-441524 plasma concentration accumulation in cases of renal impairment, which was also observed in our case. However, despite this, no significant correlation was found between serum GS-441524 concentration and the occurrence of adverse effects. In this particular case, we suspect remdesivir toxicity as the potential cause of the transient lactic acidosis and elevation of liver enzymes, possibly induced by mitochondrial toxicity.

Although experimental studies on remdesivir's potential mitochondrial toxicity have yielded conflicting results, some data do suggest its potential for causing mitochondrial toxicity. In our observation, the direct involvement of SARS-CoV-2 infection appears unlikely, as a close temporal relationship was noted between the introduction of remdesivir and the elevation of serum lactate and AST levels. Additionally, type A lactic acidosis is unlikely due to the absence of an apparent cause of shock or significant hypoxic episodes. Therefore, considering the exclusion of reduced lactate hepatic clearance as a cause (as liver enzyme changes followed the elevation of serum lactate concentration) and further supported by the development of hypoglycemia and ketonuria, type B lactic acidosis due to mitochondrial toxicity appears to be the most probable cause.

While remdesivir has been widely used for the treatment of COVID-19, its potential adverse effects, particularly in patients with renal impairment, warrant further exploration.

## Figures and Tables

**Table 1:** Medication list over the last week preceding the development of lactic acidosis.

Remdesivir	Fluconazole
Tacrolimus	Ursodeoxycholic acid
Mycophenolic acid	Piperacillin/tazobactam
Aciclovir	Pantoprazole

## Data Availability

Data regarding this patient case and pharmacokinetics are stored in a secured server in our institution and are available upon request.
